# Fall Prevention Intervention in the Emergency Department for Older Adults

**Published:** 2020-04-06

**Authors:** Cameron J. Gettel, Elizabeth M. Goldberg

**Affiliations:** 1 Department of Emergency Medicine, Yale University School of Medicine, New Haven, USA; 2 Department of Internal Medicine, Yale University School of Medicine, New Haven, USA; 3 Department of Health Services, Policy and Practice, Brown University School of Public Health, Providence, Rhode Island, USA; 4 Department of Emergency Medicine, The Warren Alpert Medical School of Brown University, Providence, Rhode Island, USA

## COMMENTARY

In many emergency departments (EDs) around the world, providers care for patients who have experienced injuries related to a fall. Approximately 30% of older adults fall each year [1], resulting in serious injuries, decreased mobility, and loss of independence [2]. The annual Medicare costs for older adult falls is estimated at $31.3 billion and only expected to increase as the population continues to age [3].

### ED-based fall prevention is understudied

Falls are a major research priority for EDs, and our objective was to evaluate how patients with falls and caregivers perceive fall prevention efforts that are initiated in the ED. To help standardize care for older adults, multidisciplinary specialty societies recently created the Geriatric Emergency Department guidelines [4]. In addition to an initiative supported by the West Health Institute and the John A. Hartford Foundation, these guidelines led to an accreditation program for geriatric emergency departments. Accreditation materials include screening protocols, assessments, structure, staff education, and quality standards [5]. Accredited geriatric emergency departments vary in the staffing composition, with some employing an interdisciplinary team of social workers, physical therapists, occupational therapists, case managers, geriatrics consultants (physician or APP), and pharmacists as needed [6]. Despite fall assessments being a key criterion for accreditation, no standardized model exists to guide the care [7] or determine the necessary team members to evaluate older adults after a fall. The original qualitative investigation by Goldberg et al. addresses this topic and is an important contribution in determining optimal post-acute fall prevention intervention strategies [8].

### A new model of care for ED-based fall prevention

**Figure 1:**
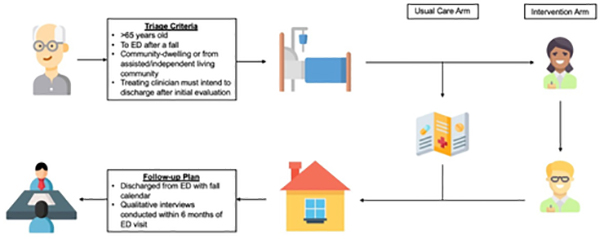
Schematic of GAPcare randomization procedures and follow-up. Usual Care Arm participants received the standard evaluation and care from the ED providers, in addition to a brochure from Centers for Disease Control and Prevention (CDC) about home safety. The Intervention Arm participants received a pharmacy and physical therapy consultation in the ED prior to discharge.

GAPcare (the Geriatric Acute and Post-acute Fall Prevention Intervention) is a randomized single-blinded pilot study involving older adults (≥ 65 years old) who presented to 1 of 2 academic US EDs after a fall. In addition to standard emergency care, participants were randomly assigned to receive usual care (control) or a pharmacist and physical therapist (PT) evaluation (intervention) [9]. We subsequently conducted in-depth interviews with patients and caregivers to determine their lived experience with the intervention as well as barriers to completing fall prevention interventions (Figure 1).

## OUR FINDINGS

We identified five overarching themes from the qualitative evaluations: (1) experiences with and receptivity to the pharmacy/PT consultations in the ED, (2) barriers to uptake of pharmacy/PT consultations and recommendations, (3) content of the consultation, (4) perceived impact of the consultations, and (5) suggestions for improvement of GAPcare. Patients noted benefits of the pharmacists in identifying reasons for medication nonadherence and subsequently simplifying the medication regimen. Caregivers, notably for patients with cognitive impairment, and patients were receptive of these changes, specifically if medications were believed to contribute to the fall. Physical therapists were also well received in providing valuable mobility feedback to patients, often starting a dialogue as to appropriate level of care (e.g. home, hospital, assisted living facility) based on functional abilities. Patients and caregivers also welcomed advice from physical therapists on how to improve safety and overcome fears of mobility and repeat falls [8].

Aside from the necessary injury assessment, more consideration must be shifted towards identifying reasons for the fall and providing fall prevention resources in the ED and/or on discharge. Currently, many fall risk assessment and fall prevention intervention strategies are multimodal, and therefore difficult to identify the effect of individual components. Further research must be conducted to screen and identify specific personnel evaluations and interventions that older adults may benefit from after a fall. Once an intervention is identified, the challenges of its implementation must be considered. Many older adults do not acknowledge the importance of falls and wish to avoid the stigma associated with what may be a ‘sentinel event’ [7]. The population of older adults who have fallen is also more likely to have cognitive impairment, similarly making intervention delivery challenging.

## FUTURE DIRECTIONS

Three key considerations for developing fall prevention programs in the ED include:

- Consider patient and caregiver concerns over timeliness and burden. Patients already spend on average 4.5 hours in US EDs and older adults are more susceptible to poor outcomes with lengthy ED stays.
- Patients and families require education about the importance of fall prevention. ED visits can be teachable moments, but barriers to prevention interventions include concerns over stigma, cost, and burden of interventions.
- In a resource-limited setting, consider implementation in high-risk patients. For example, older adults with cognitive impairment have an increased risk of falling and are frequently excluded from trials and interventions.

Furthermore, ED-based physical therapy, in particular, has been shown to be effective in reducing subsequent fall-related ED visits [10]. PT assessments and referrals remain rare within EDs despite these findings. In accordance with the geriatric emergency department guidelines and accreditation programs, not every older adult requires a comprehensive assessment in the ED. However, It is imperative that studies evaluate which members of the team will improve patient-centered outcomes and which high-risk patients may benefit from a comprehensive interdisciplinary team. Incorporating these evaluations into the clinical ED flow while considering financial and time constraints will be necessary for interventions to prove beneficial.
